# Supplementary material for: A modular lentiviral system for multiplexed gene perturbation and functional analysis suggests interdependence of hormone receptors in breast cancer growth in vivo
Source: Life Sci Alliance. 2026 Nov 5;10(1):e202603911. doi: 10.26508/lsa.202603911 (PMC13740142; doi:10.26508/lsa.202603911)
Supplement: Supplementary file 1 [file LSA-2026-03911_review_history.pdf]

Multiplex lentiviral gene perturbation suggests hormone receptor crosstalk in breast cancer in vivo

Seher Alam, Daria Matvienko, Johan Boström, Maria Pires, Aikaterini Papadimitropoulou, Saeed Eshtad, Kumar Sanjiv, Hong Qian, Nicholas Valerie, Cathrin Brisken, and Mikael Altun
DOI: https://doi.org/10.26508/lsa.202603911

Corresponding author(s): Mikael Altun, Karolinska Institutet and Cathrin Brisken, EPFL

Review Timeline:

Submission Date:	2026-09-16
Editorial Decision:	2026-10-06
Revision Received:	2026-10-13
Accepted:	2026-10-20

Scientific Editor: Sarita Hebbar

Transaction Report:

(Note: With the exception of the correction of typographical or spelling errors that could be a source of ambiguity, letters and reports are not edited. The original formatting of letters and referee reports may not be reflected in this compilation.)

Please note that the manuscript was reviewed at *Review Commons* and these reports were taken into account in the decision-making process at *Life Science Alliance.*

Reviews

Review #1

1. Evidence, reproducibility and clarity:

Summary:

The manuscript describes a modular, doxycycline-inducible lentiviral vector platform that enables conditional overexpression, RNAi-mediated knockdown, and CRISPR-based perturbations, combined with fluorescent and luminescent reporters for multiplexed tracking of cell populations. Using this system, the authors perform pooled, competitive in vitro and in vivo assays, focusing on hormone receptor dependencies (ER, PR, AR) in MCF7 breast cancer cells. The key biological conclusion is that AR depletion has minimal effects in vitro but significantly impairs growth in vivo, and that combined hormone receptor knockdown leads to synergistic growth suppression in a xenograft model.

Major comments:

1. Strength of evidence supporting the key conclusions: The technical demonstration of the vector platform is generally convincing, particularly the modular design and the feasibility of multiplexed fluorescent tracking. However, the biological conclusions are only partially supported by the data. The claim that hormone receptor interdependence, and in particular AR dependence, is revealed specifically in vivo rests on a single cell line (MCF7), a limited number of animals, and a small set of shRNAs-some of which appear to have weak or no functional impact in vitro. As such, the conclusions should be clearly qualified as preliminary and context-specific, rather than presented as generalizable insights into hormone receptor biology.

In particular, the strong concluding statements (final paragraph of the manuscript) should be toned down to reflect: the limited number of models tested, the absence of mechanistic insight, and the reliance on RNAi-based perturbations without rescue experiments.

2. Claims that require qualification or revision: Several claims appear overstated relative to the data provided:

a. The assertion that the system enables robust temporal control of perturbations is not supported by quantitative data on leakiness, induction kinetics, or stability of editing over time.

b. The claim that pooled multiplexed perturbation "reveals" discrepancies between in vitro and in vivo AR function is based on a narrow experimental scope and should be reframed as an illustrative example rather than a definitive finding.

c. Statements implying reduced off-target effects due to temporal regulation are speculative and should be removed unless supported by data.

3. Additional experiments essential to support the paper (limited and realistic): Only minimal additional experiments are required to support the manuscript as it stands:

a. Quantification of inducibility and leakiness of the dual Tet CRISPR system (e.g., untreated vs. dox-treated control in Fig. 2C, and time-course of editing efficiency).

b. Quantification of FUCCI reporter outputs (cell-cycle phase distributions) rather than representative images alone.

4. Reproducibility and methodological clarity: Several aspects of the methodology require clarification to ensure reproducibility:

a. Lentiviral titers and recombination rates are not reported. Given the complexity and size of the constructs, this information is essential.

b. It is unclear how background editing is prevented in the dual Tet CRISPR system, since both Cas9 and gRNA are present in the same cells and may exhibit basal expression.

c. The manuscript does not adequately address integration-based leakiness of doxycycline-inducible systems in lentiviral backbones, especially compared to transposon-based approaches.

d. The description of how MCF7-luciferase cells are used to generate lentiviral vectors is confusing and must be clarified.

5. Replication and statistical analysis: The statistical treatment of pooled competition data is insufficiently detailed. It is unclear how many animals, glands, or technical replicates contribute to each comparison. The manuscript does not clearly report the fraction of cells recovered for single, double, and triple knockdowns. Multiple comparisons and normalization strategies are not consistently explained.

Minor comments:

a. Comparison to existing Gateway-compatible systems (e.g. the John Doench system) would help contextualize the technical advance.

b. Page 5, line 6: "The ..." should be "It ...".

c. P2A sequences are not self-cleaving; the manuscript should correctly state that the ribosome skips peptide bond formation between the last two amino acids.

d. Fig. 2C lacks an untreated control.

e. Fig. 2E (AkaLuc panel): the label "Reagent" is incorrect and overly broad; a clearer and more consistent nomenclature is needed.

f. The purpose of fluorescence-based tracking of cell populations is not clearly explained; the rationale should be explicitly stated.

g. Claims that hormone supplementation rescues AR depletion are not supported, particularly given the lack of a clear AR-dependent phenotype in prior assays (e.g. Fig. S3A).

h. The large difference in proliferation between NT cells {plus minus} doxycycline in Fig. 4B is concerning and may reflect a normalization or analysis error; this should be addressed.

i. Figures would benefit from clearer legends specifying n, statistical tests, and normalization procedures.

2. Significance:

Nature and significance of the advance: This work represents a technical advance, rather than a conceptual or biological one. The modular lentiviral platform for inducible perturbations and multiplexed fluorescent tracking is potentially useful, particularly for pooled in vivo competition assays where reducing animal use is desirable.

Context within existing literature: Inducible lentiviral shRNA and CRISPR systems, as well as fluorescent barcoding strategies, are well established. The main contribution here is the integration of these elements into a single, modular framework. However, the manuscript would benefit from clearer comparison to existing systems (including Gateway-based and inducible CRISPR platforms) and discussion of known limitations of lentiviral Tet systems.

Audience: The work will primarily interest I) cancer biologists performing functional genetic screens, ii) researchers developing or applying genetic perturbation tools, and iii) laboratories interested in pooled in vivo assays. The biological findings regarding hormone receptor interdependence are likely of more limited interest unless further validated.

Reviewer expertise: My expertise includes functional cancer genomics, lentiviral and CRISPR-based perturbation systems, in vitro and in vivo genetic screening approaches, as well as bioinformatics. I have sufficient expertise to evaluate the technical platform and biological conclusions presented.

3. How much time do you estimate the authors will need to complete the suggested revisions:

(Decision Recommendation)

Between 1 and 3 months

4. Review Commons values the work of reviewers and encourages them to get credit for their work. Select 'Yes' below to register your reviewing activity at Web of Science Reviewer Recognition Service (formerly Publons); note that the content of your review will not be visible on Web of Science.

Web of Science Reviewer Recognition

Yes

Review #2

1. Evidence, reproducibility and clarity:

Summary

This study presents a novel, modular lentiviral platform integrating inducible overexpression, shRNA knockdown, and CRISPR/Cas9 editing within a single system. Its core innovation lies in the versatile design, utilizing Gateway cloning to enable rapid exchange of 14 fluorescent proteins, 3 luminescent reporters, and selection markers, facilitating high-content phenotyping. A dual doxycycline-inducible architecture ensures precise temporal control, minimizing off-target effects. Methodologically, it introduces a robust fluorescence barcoding strategy, allowing multiplexed tracking of up to nine distinct genetic perturbations in pooled assays. When applied to breast cancer intraductal xenografts, this revealed critical in vivo receptor interdependencies-such as the essential roles of AR, ER and PR, in tumor growth. By enabling combinatorial genetic analysis within single animals, this technology significantly reduces experimental variability and animal usage by up to eightfold, advancing both the efficiency of functional genomics and adherence to ethical research standards.

Major comments

1. Despite using an inducible system, shRNA and CRISPR components may still have off-target effects. In the F4 study, was whole-genome sequencing or transcriptomic analysis performed to assess unintended perturbations of non-target genes?

2. Barcode stability: Are the fluorescent barcodes stably expressed during long-term in vivo culture? Is there a risk of silencing or loss that could affect the reliability of long-term tracking?

3. Cell-cell interference: In mixed transplantation experiments, could different genotypes influence each other through paracrine signaling or competition for resources, leading to observed growth phenotypes that are not entirely cell-autonomous?

4. Applicability to non-coding genes or weak-effect genes: This platform relies on observable phenotypic changes for screening. Is it sensitive enough for genes with weak effects or functional redundancy in regulatory networks?

5. Are there differences in the induction efficiency of different shRNA or CRISPR components? Could this lead to biases in certain barcode signals, thereby affecting the accuracy of competitive growth analysis?

6. Are there variations in the packaging efficiency of different shRNA or CRISPR components into lentiviral vectors? Does this affect viral titer? How is copy number consistency achieved in cells after lentiviral transduction? Has the knockdown efficiency been compared between cells transduced with 3 shRNAs and those with single shRNA?

Minor comments

1. The labeling "NT" in Data F3 and Supplementary Data SF3 is unclear. Do they refer to the same condition? Is DOX added in the "NT" condition in SF3?

2. Cost-effectiveness ratio: Although animal use is reduced, is the cost of constructing the multiplexed barcoded viral library significantly higher than traditional methods? Is its overall economic feasibility suitable for large-scale screening?

2. Significance:

The most important significance of the study is integrating multiple technologies into one system enabling high-content phenotyping, which may facilitate the discovery of new biological pathways.

3. How much time do you estimate the authors will need to complete the suggested revisions:

Estimated time to Complete Revisions (Required)

(Decision Recommendation)

Between 1 and 3 months

4. Review Commons values the work of reviewers and encourages them to get credit for their work. Select 'Yes' below to register your reviewing activity at Web of Science Reviewer Recognition Service (formerly Publons); note that the content of your review will not be visible on Web of Science.

Web of Science Reviewer Recognition

Yes

Manuscript number: RC- 2025-03337
Corresponding author(s): Cathrin, Brisken and Mikael, Altun

[Please use this template only if the submitted manuscript should be considered by the affiliate journal as a full revision in response to the points raised by the reviewers.

If you wish to submit a preliminary revision with a revision plan, please use our "Revision Plan" template. ***It is important to use the appropriate template to clearly inform the editors of your intentions.****]*

1. General Statements [optional]

This section is optional. Insert here any general statements you wish to make about the goal of the study or about the reviews.

This manuscript describes a modular, doxycycline-inducible lentiviral platform that integrates gene overexpression, shRNA-mediated knockdown, and CRISPR/Cas9-mediated perturbation within a single system, combined with fluorescence and luminescence multiplexing for tracking pooled cell populations. Using this platform, we investigate hormone receptor (ER, PR, AR) interdependence in MCF7 breast cancer cells, both in vitro and in intraductal xenografts in vivo, and show that combinatorial hormone receptor knockdown produces additive-to-synergistic growth suppression while substantially reducing animal use through pooled, barcoded competition assays.

We thank both reviewers for their constructive and detailed evaluation. Reviewer #1 raised concerns about the strength of evidence for our biological conclusions, given the reliance on a single cell line and a limited, non-exhaustively benchmarked panel of shRNAs, and requested quantitative data on the leakiness and induction kinetics of our dual doxycycline-inducible CRISPR/Cas9 system. Reviewer #2 focused on technical aspects of the platform, including barcode stability, packaging efficiency, and how differences in induction efficiency between constructs might bias the pooled competition assay.

In this full revision, we have addressed all reviewer comments, as detailed in the point-by-point response below. Briefly, we have: (i) added a caveat paragraph explicitly tempering the biological conclusions as preliminary and context-specific, and softened the manuscript's closing statement; (ii) generated new quantitative data on the induction kinetics and background editing rate of the dual-inducible CRISPR/Cas9 system by long-read (ONT) amplicon sequencing (new Fig. 2G), and quantified our FUCCI cell-cycle reporter (new Fig. 3); (iii) added two new Discussion paragraphs contextualizing our platform relative to existing Gateway-based and CRISPR/Cas9 screening resources, and addressing integration-site-dependent leakiness of doxycycline-inducible lentiviral systems relative to transposon-based approaches; (iv) expanded our in vivo competition assay analysis to normalize each doxycycline-treated barcoded

population to its own untreated baseline before comparison to the non-targeting control, thereby controlling for differences in engraftment between populations; and (v) clarified the rationale for fluorescence-based barcoding, corrected the description of P2A-mediated ribosomal skipping, and addressed several additional minor points raised by both reviewers. We believe the revised manuscript directly and substantively addresses the reviewers' concerns.

2. Point-by-point description of the revisions

This section is mandatory. Please insert a point-by-point reply describing the revisions that were ***already carried out and included*** *in the transferred manuscript.*

Reviewer #1 (Evidence, reproducibility and clarity (Required)):

Summary:
The manuscript describes a modular, doxycycline-inducible lentiviral vector platform that enables conditional overexpression, RNAi-mediated knockdown, and CRISPR-based perturbations, combined with fluorescent and luminescent reporters for multiplexed tracking of cell populations. Using this system, the authors perform pooled, competitive in vitro and in vivo assays, focusing on hormone receptor dependencies (ER, PR, AR) in MCF7 breast cancer cells. The key biological conclusion is that AR depletion has minimal effects in vitro but significantly impairs growth in vivo, and that combined hormone receptor knockdown leads to synergistic growth suppression in a xenograft model.

Major comments:
1. Strength of evidence supporting the key conclusions: The technical demonstration of the vector platform is generally convincing, particularly the modular design and the feasibility of multiplexed fluorescent tracking. However, the biological conclusions are only partially supported by the data. The claim that hormone receptor interdependence, and in particular AR dependence, is revealed specifically in vivo rests on a single cell line (MCF7), a limited number of animals, and a small set of shRNAs-some of which appear to have weak or no functional impact in vitro. As such, the conclusions should be clearly qualified as preliminary and context-specific, rather than presented as generalizable insights into hormone receptor biology.
In particular, the strong concluding statements (final paragraph of the manuscript) should be toned down to reflect: the limited number of models tested, the absence of mechanistic insight, and the reliance on RNAi-based perturbations without rescue experiments.
We thank the reviewer for this important point. We agree that reliance on a single cell line, limited animal numbers, and possible variability in shRNA performance warranted more cautious framing of the biological conclusions. We have added an explicit caveat paragraph before the closing summary stating that these findings should be regarded as proof of concept, generated in a single breast cancer cell line (MCF7) using a limited number of animals and a small panel of shRNAs whose individual potency was not exhaustively benchmarked. The AR-dependency finding, in particular, is based on RNA interference alone, without orthogonal rescue experiments or

mechanistic follow-up, and should be interpreted as preliminary and context-specific rather than as a generalizable feature of AR biology. We have also softened the closing sentence of the manuscript from "accelerates the preclinical development..." to "may help inform future preclinical strategies for hormone-sensitive breast cancer and beyond, pending validation in additional models."

2. Claims that require qualification or revision: Several claims appear overstated relative to the data provided:
a. The assertion that the system enables robust temporal control of perturbations is not supported by quantitative data on leakiness, induction kinetics, or stability of editing over time.
Thank you for raising these points. To address the concerns, we repeated the experiments, including time-course analyses and DNA sequencing with the inducible CAS9 and qRNA, and now present the data in Figure 2. These results show the induction kinetics of the system and address the leakiness of the double-inducible CRISPR/CAS9 system. Because DNA editing is a permanent change to the DNA, we assume that the stability of the edit over time will also be permanent (see Figure 2 and Supplemental Table 2).

b. The claim that pooled multiplexed perturbation "reveals" discrepancies between in vitro and in vivo AR function is based on a narrow experimental scope and should be reframed as an illustrative example rather than a definitive finding.
Thank you for pointing this out. We have revised and softened the claims by removing "reveals" and replacing it with "suggests" throughout the manuscript.

c. Statements implying reduced off-target effects due to temporal regulation are speculative and should be removed unless supported by data.
Thank you for pointing this out. We have removed those parts from the manuscript and revised the relevant sentences to avoid speculation about off-target effects, which are largely determined by the design of gRNAs and shRNAs.

3. Additional experiments essential to support the paper (limited and realistic): Only minimal additional experiments are required to support the manuscript as it stands:
a. Quantification of inducibility and leakiness of the dual Tet CRISPR system (e.g., untreated vs. dox-treated control in Fig. 2C, and time-course of editing efficiency).
Thank you for the suggestion. We have now performed this experiment and included the data (see the new Figure 2G).

b. Quantification of FUCCI reporter outputs (cell-cycle phase distributions) rather than representative images alone.
Thank you for the suggestion. We repeated the experiment and quantified the FUCCI reporter output (see Figure 3).

4. Reproducibility and methodological clarity: Several aspects of the methodology require

clarification to ensure reproducibility:
a. Lentiviral titers and recombination rates are not reported. Given the complexity and size of the constructs, this information is essential.
We have added more detailed descriptions of the experimental procedures to the Methods section and have repeated the Cas9 transduction experiments, including their corresponding results and viral titers (see Figure 2 and Supplementary Figure 6).

b. It is unclear how background editing is prevented in the dual Tet CRISPR system, since both Cas9 and gRNA are present in the same cells and may exhibit basal expression.
We use serum from South America, where standard antibiotic treatment is less common than in the United States, reducing the likelihood that doxycycline is present and minimizing basal expression. The rationale for using double-inducible systems is to minimize basal expression of both components and thereby reduce the chance that either one reaches levels sufficient for editing. We have also made the underlying design logic explicit in the manuscript: because unwanted editing requires both Cas9 and gRNA to be simultaneously present above a functional threshold, and each is independently repressed through a distinct tetracycline-responsive mechanism (Tet-On for Cas9, Tet-Off for gRNA), the probability of coincident leaky expression of both components is substantially lower than for either component alone. This is now linked to the empirically measured background editing rate (~4% over 11 days without doxycycline, by ONT sequencing; Fig. 2G).

c. The manuscript does not adequately address integration-based leakiness of doxycycline-inducible systems in lentiviral backbones, especially compared to transposon-based approaches.
Thanks for this point. While we have not directly compared our system with transposon-based approaches, we have added a brief section on this in the Discussion to address this point explicitly. Because our system relies on polyclonal, antibiotic- or FACS-selected populations rather than single-cell-validated clones, some degree of integration site–dependent leakiness in the noninduced state cannot be fully excluded and may contribute to the low background editing (~4%) we observe over extended culture. We contrast this with transposon-based delivery systems (piggyBac, Sleeping Beauty), which have distinct genomic integration profiles relative to lentivirus, and note that doxycycline-inducible piggyBac constructs have, in some contexts, achieved tighter regulation with undetectable basal leakiness *in vivo*. This may reflect the absence of viral LTR-associated regulatory elements and the lentiviral bias toward active chromatin. We discuss chromatin insulators, safe-harbor-targeted integration, and transposon-based delivery as potential strategies to further reduce leaky background expression in future iterations of the platform.

d. The description of how MCF7-luciferase cells are used to generate lentiviral vectors is confusing and must be clarified.
We have updated this in the revised manuscript and hope it is clearer now.

5. Replication and statistical analysis: The statistical treatment of pooled competition data is insufficiently detailed. It is unclear how many animals, glands, or technical replicates contribute to

each comparison. The manuscript does not clearly report the fraction of cells recovered for single, double, and triple knockdowns. Multiple comparisons and normalization strategies are not consistently explained.
We apologize if these sections were difficult to follow and have substantially expanded them in the manuscript. Because a recurring concern with any pooled, barcoded competition assay is that the fluorescent tag, lentiviral integration site, or clonal origin of a population could itself influence engraftment or proliferation independently of the intended genetic perturbation, we now normalize each doxycycline-treated population to its own untreated (−Dox) baseline and then express it relative to the equivalently normalized NT population within the same tumor (Fig. 5C, F). We also report the numbers of animals and glands contributing to each comparison directly in the Fig. 5 legend, and we justify this normalization approach in more detail in a new paragraph in the Discussion.

Minor comments:
a. Comparison to existing Gateway-compatible systems (e.g. the John Doench system) would help contextualize the technical advance.
Thank you. We have now added a paragraph to the Discussion that compares our platform with existing Gateway-based and CRISPR/Cas9 lentiviral resources, including the Broad Institute's Genetic Perturbation Platform co-developed by Doench and colleagues (Brunello, Dolcetto, Calabrese). This paragraph clarifies that our system is complementary to these genome-scale discovery tools rather than competing with them: it is designed for hypothesis-driven, combinatorial, multiplexed perturbation studies with tight temporal control and validated *ex vivo* and *in vivo* applications, rather than for large-scale single-modality screening.

b. Page 5, line 6: "The ..." should be "It ...".
Thank you for the comment and we have corrected this.
c. P2A sequences are not self-cleaving; the manuscript should correctly state that the ribosome skips peptide bond formation between the last two amino acids.
Thank you for the comment and we have corrected this.
d. Fig. 2C lacks an untreated control.
Thank you for this. Instead of using an untreated control, we used two different guides targeting USP14 and USP7 to validate the inducible gRNA with constitutively expressed Cas9. For the knockdown controls, each gene served as the control for the other in the corresponding experiments, demonstrating that knockdown could be achieved with the specific inducible gRNA.

e. Fig. 2E (AkaLuc panel): the label "Reagent" is incorrect and overly broad; a clearer and more consistent nomenclature is needed.
Thank you for bringing this to our attention; we have corrected it in the revised manuscript. The panel has also been moved and now appears as Fig. 3B, with the axis relabeled "Ctrl"/"+Substrate" in place of "Reagent."
f. The purpose of fluorescence-based tracking of cell populations is not clearly explained; the rationale should be explicitly stated.

We have now added an explicit rationale at the start of this Results subsection: multiplexed fluorescence barcoding is used to pool several genetically distinct cell populations and track them side by side within the same well, culture, or animal. This allows different genotypes to be compared under identical experimental conditions rather than across separate parallel experiments. This internally controlled design reduces confounding variability arising from well-to-well, batch-to-batch, or animal-to-animal differences and increases the statistical power obtained per experiment. It is particularly valuable *in vivo*, where it substantially reduces the number of animals required, in keeping with the 3Rs principles.

g. Claims that hormone supplementation rescues AR depletion are not supported, particularly given the lack of a clear AR-dependent phenotype in prior assays (e.g. Fig. S3A).
Thank you for bringing this up. We have rewritten this section and clarified that it behaves more like the non-targeting control shRNA (NT).

h. The large difference in proliferation between NT cells {plus minus} doxycycline in Fig. 4B is concerning and may reflect a normalization or analysis error; this should be addressed.
Thank you for pointing this out. We have now included additional raw data for clarity and addressed the issue accordingly, so it should not be misinterpreted in the future.
i. Figures would benefit from clearer legends specifying n, statistical tests, and normalization procedures.
Thank you for pointing this out; we have addressed it in the revised manuscript.
Reviewer #1 (Significance (Required)):

Nature and significance of the advance: This work represents a technical advance, rather than a conceptual or biological one. The modular lentiviral platform for inducible perturbations and multiplexed fluorescent tracking is potentially useful, particularly for pooled in vivo competition assays where reducing animal use is desirable.

Context within existing literature: Inducible lentiviral shRNA and CRISPR systems, as well as fluorescent barcoding strategies, are well established. The main contribution here is the integration of these elements into a single, modular framework. However, the manuscript would benefit from clearer comparison to existing systems (including Gateway-based and inducible CRISPR platforms) and discussion of known limitations of lentiviral Tet systems.

Audience: The work will primarily interest I) cancer biologists performing functional genetic screens, ii) researchers developing or applying genetic perturbation tools, and iii) laboratories interested in pooled in vivo assays. The biological findings regarding hormone receptor interdependence are likely of more limited interest unless further validated.

Reviewer expertise: My expertise includes functional cancer genomics, lentiviral and CRISPR-based perturbation systems, in vitro and in vivo genetic screening approaches, as well as bioinformatics. I have sufficient expertise to evaluate the technical platform and biological

conclusions presented.

Reviewer #2 (Evidence, reproducibility and clarity (Required)):

Summary
This study presents a novel, modular lentiviral platform integrating inducible overexpression, shRNA knockdown, and CRISPR/Cas9 editing within a single system. Its core innovation lies in the versatile design, utilizing Gateway cloning to enable rapid exchange of 14 fluorescent proteins, 3 luminescent reporters, and selection markers, facilitating high-content phenotyping. A dual doxycycline-inducible architecture ensures precise temporal control, minimizing off-target effects. Methodologically, it introduces a robust fluorescence barcoding strategy, allowing multiplexed tracking of up to nine distinct genetic perturbations in pooled assays. When applied to breast cancer intraductal xenografts, this revealed critical in vivo receptor interdependencies-such as the essential roles of AR, ER and PR, in tumor growth. By enabling combinatorial genetic analysis within single animals, this technology significantly reduces experimental variability and animal usage by up to eightfold, advancing both the efficiency of functional genomics and adherence to ethical research standards.

Major comments
1. Despite using an inducible system, shRNA and CRISPR components may still have off-target effects. In the F4 study, was whole-genome sequencing or transcriptomic analysis performed to assess unintended perturbations of non-target genes?
Because we did not aim to conduct detailed mechanistic studies or present shRNA as a new technology, and because the hairpins used are not novel and have already been described in published studies, we did not attempt to identify or test potential off-target genes in this work.

2. Barcode stability: Are the fluorescent barcodes stably expressed during long-term in vivo culture? Is there a risk of silencing or loss that could affect the reliability of long-term tracking?
The *in vivo* studies we conducted lasted more than 30 days. The cells were first validated by qPCR and Western blot, generating transgenic cells that were then expanded for all replicates and validations, including the *in vivo* experiments. The barcodes are driven by the EF1α promoter, which is not expected to be susceptible to methylation and, in theory, should support expression in long-term *in vivo* studies even beyond the 30-day duration used here.

3. Cell-cell interference: In mixed transplantation experiments, could different genotypes influence each other through paracrine signaling or competition for resources, leading to observed growth phenotypes that are not entirely cell-autonomous?
Thank you for raising this important point. We observed similar *ex vivo* effects on growth reduction in single experiments (except for AR *in vitro*) as we did *in vivo* with the mixed population. In all *in vivo* studies, whether mixed or single, the cancer cells injected into a mouse are never cell-autonomous. That is also why one performs *in vivo* studies using genetic perturbations: to

demonstrate that cellular dependencies on genes are not *in vitro* artifacts, to show that certain genes are important in an *in vivo* setting, and to reveal how a gene affects the microenvironment *in vivo*, not just *ex vivo*. Because tumors are inherently heterogeneous, one could also argue that a mixture of different genotypes allows us to study tumor evolution with greater insight into different gene losses.

4. Applicability to non-coding genes or weak-effect genes: This platform relies on observable phenotypic changes for screening. Is it sensitive enough for genes with weak effects or functional redundancy in regulatory networks?
Thank you for the interesting question. In theory, the approach should be sensitive enough to use with genes that do not cause a growth defect. The functional readouts should then be further tailored to this purpose, using reporter assays, biomarker assays, or alternative sequencing-based readouts to study different gene-specific consequences. The barcodes can still be used to separate cells by flow cytometry and then to analyze them with the assay of choice.

5. Are there differences in the induction efficiency of different shRNA or CRISPR components? Could this lead to biases in certain barcode signals, thereby affecting the accuracy of competitive growth analysis?
Thank you for the question. We did not detect any induction efficiency effects that would bias the barcoding, as we used hairpins and gRNA guides that have been validated or previously used in the literature. However, this does not mean such bias could not occur in some cases.
Because we also use a "no doxycycline" control, one can not only study the intercell grafting efficacy of any transgenic cell but also estimate the theoretical growth based on NT and non-dox samples. This can normalize any calculations if such bias affects the dynamics of the hairpin or gRNA. Inclusion of the no-dox control accounts for this directly: each doxycycline-treated population is normalized to its own untreated (−Dox) baseline before being expressed relative to the similarly normalized NT population within the same tumor (Fig. 5C, F). We have now implemented and reported this normalization in the manuscript, with the rationale explained in a new Discussion paragraph, rather than treating it only as a theoretical mitigation.

6. Are there variations in the packaging efficiency of different shRNA or CRISPR components into lentiviral vectors? Does this affect viral titer? How is copy number consistency achieved in cells after lentiviral transduction? Has the knockdown efficiency been compared between cells transduced with 3 shRNAs and those with single shRNA?
Thank you for the questions. For the shRNA and gRNA transductions, the plasmids are approximately the same size, and we have not observed any differences in transduction efficiency between these vectors. Purity and other factors during vector isolation usually have a more pronounced effect on transduction efficiency.
The vectors that are difficult to transduce are those encoding CAS9 itself; in particular, inducible CAS9 is more challenging to transduce, and achieving a good viral titer is important for efficient transduction. We repeated an experiment and transduced the cells with different viral titers and

found that the number of cells surviving antibiotic selection correlated with titer, but after expansion they expressed similar levels of CAS9.
Since we do not use monoclonal cells and instead work with polyclonal transgenic lines, we assume that random integration should occur in a similar way as in the NT control, and therefore we did not karyotype the cells or analyze copy numbers. The knockdown efficiency was similar when comparing single and triple knockdown. We have now incorporated this explanation directly into the Methods section of the manuscript, stating explicitly that all shRNA and gRNA expression plasmids are of comparable size with no consistent differences in packaging or transduction efficiency, that titer is more strongly influenced by plasmid purity and preparation than by insert identity, and that knockdown efficiency assessed by Western blot was comparable between single, double, and triple knockdown lines (Fig. 4E–G), indicating that combinatorial transduction with multiple shRNAs did not measurably compromise silencing efficiency per target.

Minor comments
1. The labeling "NT" in Data F3 and Supplementary Data SF3 is unclear. Do they refer to the same condition? Is DOX added in the "NT" condition in SF3?
Thank you for pointing this out. NT refers to non-targeting shRNA cells, and they are treated the same as the other cells in all panels. We have updated the figure legend to clarify this.
2. Cost-effectiveness ratio: Although animal use is reduced, is the cost of constructing the multiplexed barcoded viral library significantly higher than traditional methods? Is its overall economic feasibility suitable for large-scale screening?
The cost of creating any shRNA or gRNA is only negligibly higher when generating different guides or hairpins and producing them with multiple barcodes, especially compared with *in vivo* study and animal costs. That is why we based our calculations not on construct costs but on animal costs and, more importantly, on the reduction in the number of animals needed.
For large-scale screening, the system can distinguish only among nine barcodes, gene targets, and all their combinations, so it is not currently designed for settings in which more than nine genes are targeted.

Reviewer #2 (Significance (Required)):

The most important significance of the study is integrating multiple technologies into one system enabling high-content phenotyping, which may facilitate the discovery of new biological pathways.

October 6, 2026

RE: Life Science Alliance Manuscript #LSA-2026-03911-T

Mikael Altun
Karolinska Institute
Science for Life Laboratory, Division of Translational Medicine and Chemical Biology, Department of Medical Biochemistry and Biophysics
Tomtebodavägen 23 B
Stockholm 17165
Sweden

Dear Dr. Altun,

Thank you for submitting your revised manuscript entitled "Multiplex lentiviral gene perturbation suggests hormone receptor crosstalk in breast cancer in vivo".

Your manuscript was assessed by Reviewer 1 who has noted an improvement in the revised version having addressed their concerns. In light of the overall positive evaluation, we would be happy to publish your paper in Life Science Alliance pending resolution of the below mentioned points raised by this reviewer, and final revisions necessary to meet our formatting guidelines.
-check for language clarity and use of appropriate terminology (points 1 and 3).
-appropriate citation for shRNA vector architecture (point 2).

Please submit a manuscript with changes highlighted and a point-by-point response to the reviewers comments.

MANUSCRIPT ORGANIZATION AND FORMATTING:

To avoid unnecessary delays in the acceptance and publication of your paper, please read the following information carefully. Full guidelines are available on our Instructions for Authors page, https://www.life-science-alliance.org/authors

Submission of a paper that does not conform to Life Science Alliance guidelines will delay the acceptance of your manuscript.

-Please add scale bars and size information for Figures 3D and 4C.
-Please include molecular weight markers for Figure 2C.
-Please specify concentration for antibodies used in this work.
-Kindly expand on the details of imaging methods by including objective name/type, numerical aperture.
-Please upload your main manuscript text as an editable doc file.
-Please upload your main and supplementary figures as single files.
-Please add a Running Title in our system.
-Please add ORCID ID for the corresponding (and secondary corresponding) author--you should have received instructions on how to do so.
-Please add a Summary Blurb/Alternate Abstract in our system.
-Please add a Category for your manuscript in our system.
-Please add the X and Bluesky handles of your host institute/organization, as well as your own, and/or one of the authors, in our system.
-The titles in both the system and the manuscript file must be consistent with each other.
-Please move all sections currently provided after the references section before the references.
-Please add the authors' contributions to our system as well.
-It is recommended to exclude figures from the manuscript text and upload them separately.
-Please remove legends from your figures.
-Please add your main, supplementary figure, and table legends to the main manuscript text after the references section.
-Supplementary tables have not been uploaded.
-Please add callouts for Figure S4A-B to your main manuscript text.
-The "Data Availability" section should be placed after the Materials & Methods section. Please consult our guidelines at https://www.life-science-alliance.org/manuscript-prep#format
-Please be sure that the authorship listing and order is correct.

We encourage our authors to provide original source data, particularly uncropped/-processed electrophoretic blots and spreadsheets for the main figures of the manuscript. If you would like to add source data, we would welcome one PDF/Excel-file per figure for this information. These files will be linked as supplementary "Source Data" files.

It is Life Science Alliance policy that if requested, original data images must be made available to the editors. Failure to provide original images upon request will result in unavoidable delays in publication. Please ensure that you have access to all original data images prior to final submission.

If you are planning a press release on your work, please inform us immediately to allow informing our production team and scheduling a release date.

We welcome submissions of potential cover images for the issue of LSA in which your work would appear. If you have high quality images associated with this work, please feel free to email these, with a caption, to the journal office.

LSA encourages authors to provide a 30-60 second video where the study is briefly explained. These videos will be appear embedded with the manuscript online at Life Science Alliance, and on social media to promote the published paper and authors (for examples, see https://docs.google.com/document/d/1-UWCfbE4pGcDdcgzcmiuJl2XMBJnxKYeqRvLLrLSo8s/edit?usp=sharing). Corresponding or first-authors are welcome to submit the video. Please submit only one video per manuscript. The video can be emailed to contact@life-science-alliance.org

FINAL FILES:

To upload the final version of your manuscript, please log in to your account: https://lsa.msubmit.net/cgi-bin/main.plex
You will be guided to complete the submission of your revised manuscript and to fill in all necessary information. Please get in touch in case you do not know or remember your login name.

The following items are required for acceptance.

-- An editable version of the final text (.DOC or .DOCX) is needed for copyediting (no PDFs).

-- High-resolution figure, supplementary figure and video files uploaded as individual files: See our detailed guidelines for preparing your production-ready images, https://www.life-science-alliance.org/authors

-- Summary blurb (enter in submission system): A short text summarizing in a single sentence the study (max. 200 characters including spaces). This text is used in conjunction with the titles of papers, hence should be informative and complementary to the title. It should describe the context and significance of the findings for a general readership; it should be written in the present tense and refer to the work in the third person. Author names should not be mentioned.

The license to publish form must be signed before your manuscript can be sent to production. A link to the license to publish form will be available to the corresponding author only. Please take a moment to check your funder requirements.

Reviews, decision letters, and point-by-point responses associated with peer-review at Life Science Alliance will be published, alongside the manuscript. If you do want to opt out of having the reviewer reports and your point-by-point responses displayed, please let us know immediately.

Thank you for your attention to these final processing requirements. Please revise and format the manuscript and upload materials as soon as you are able.

Thank you for this interesting contribution to the literature. We look forward to publishing your paper in Life Science Alliance.

Sincerely,

Sarita Hebbar, PhD
Scientific Editor
Life Science Alliance
http://www.lsajournal.org

Reviewer #1 (Comments to the Authors (Required)):

The authors have addressed a large fraction of my comments, and the revision is substantially improved. My overall recommendation is minor-to-moderate revision. While I do not think further experiments are required, three issues should, however, be resolved before acceptance.

1. The claim of synergy is not supported. Applying a standard multiplicative (log-additive) null model, the double and triple knockdowns are only additive and, in one case, sub-additive. Either a formal epistasis analysis is added, or the words

"synergy"/"synergistic" should be replaced with "additive" or "further depletion" throughout the manuscript.

2. Misattribution of the shRNA vector design. The manuscript credits Cellecta with pioneering the shRNA plasmid design; this is incorrect and should be corrected (detail below).

3. Wording: The manuscript carries an unusually high density of typographical errors, particularly for a revised submission, including one unreadable sentence in the Results.

Elaboration on point 2. The shRNA vector architecture that this and essentially all subsequent systems descend from was established by Stewart et al., working with Weinberg, Sharp and Novina: Lentivirus-delivered stable gene silencing by RNAi in primary cells, RNA 9:493-501 (2003), PMID 12649500. That work introduced U6-driven hairpin cassettes delivered by lentivirus, gave rise to the pLKO.1 backbone and to The RNAi Consortium libraries, and predates Cellecta's founding by several years. Commercial suppliers, Cellecta among them, adopted and repackaged this design rather than originating it. Please correct the sentence to credit Stewart et al. (2003) as the originating design, and cite the TRC/pLKO.1 lineage or the specific commercial vector actually used as downstream implementations, if that is the intended reference. Given that this manuscript is itself a vector-resource paper, accurate attribution of prior vector design matters more than usual.

Elaboration on point 3. The revision still contains an unusual number of errors. A careful proofread is needed before acceptance.
- Results, dual-inducible CRISPR section: "provides suggeststhat background editing is effectively limited". This sentence is not readable.
- Discussion: "our platform as far as we know significantly improves multiplexing capacity", Suggest "to our knowledge, our platform improves ...".
- Results, barcoding: "individual barcodes(Fig. 3E-G)", missing space.
- Discussion: "Fucci probe cell cycle probes", duplicated word.
- Methods: "see Supplementary4", should read "Supplementary Table 4".
- Statistical Analysis and Fig. 5 legend: "Fisher's LSD post-doc test", post hoc.
- Fig. 1 legend: "the Ten-On and the Tet-Off system", Tet-On.
- Fig. 2 legend: "CRISPR7Cas9", CRISPR/Cas9.
- Fig. 2 legend, panel F: the legend for panel H refers to "the time course experiment described in F and G imaging of experiment", incomplete sentence.
- Fig. 5 legend: "knocknown", knockdown.
- Acknowledgments: the sentence beginning "We would like to thank the MedH Core Flow Cytometry facility ..." ends with a stray fragment, "Institutet.", on the following line.

October 19, 2026

RE: Life Science Alliance Manuscript #LSA-2026-03911-TR

Mikael Altun
Karolinska Institutet
Science for Life Laboratory, Division of Translational Medicine and Chemical Biology, Department of Medical Biochemistry and Biophysics
Alfred Nobels Alle 8
Stockholm 17165
Sweden

Dear Dr. Altun,

Thank you for submitting your Research Article entitled "Multiplex lentiviral gene perturbation suggests hormone receptor crosstalk in breast cancer in vivo". It is a pleasure to let you know that your manuscript is now accepted for publication in Life Science Alliance. Congratulations on this interesting work.

The final published version of your manuscript will be deposited by us to PubMed Central upon publication.

Your manuscript will now progress through copyediting and proofing. At the proofing stage, please remember to use the shorter version of the title on the manuscript submission portal as the final title for this work. The longer one in the manuscript document will not be allowed because it is substantially over the recommended length.

It is journal policy that authors provide original data upon request.

Your article will publish open access upon publication under a CC-BY license.

Reviews, decision letters, and point-by-point responses associated with peer-review at Life Science Alliance will be published, alongside the manuscript. If you do want to opt out of having the reviewer reports and your point-by-point responses displayed, please let us know immediately.

IMPORTANT: If you will be unreachable at any time, please provide us with the email address of an alternate author. Failure to respond to routine queries may lead to unavoidable delays in publication.

Scheduling details will be available from our production department. You will receive proofs shortly before the publication date. Only essential corrections can be made at the proof stage so if there are any minor final changes you wish to make to the manuscript, please let the journal office know now.

DISTRIBUTION OF MATERIALS:
Authors are required to distribute freely any materials used in experiments published in Life Science Alliance. Authors are encouraged to deposit materials used in their studies to the appropriate repositories for distribution to researchers.

You can contact the journal office with any questions, contact@life-science-alliance.org

Again, congratulations on a very nice paper. I hope you found the review process to be constructive and are pleased with how the manuscript was handled editorially. We look forward to future exciting submissions from your lab.

Sincerely,

Sarita Hebbar, PhD
Scientific Editor
Life Science Alliance
http://www.lsajournal.org
